# Dental health of children with cerebral palsy

**DOI:** 10.17712/nsj.2016.4.20150729

**Published:** 2016-10

**Authors:** Basil M. Jan, Mohammed M. Jan

**Affiliations:** *From the Department of Pediatrics, Faculty of Medicine, King Abdulaziz University, Jeddah, Kingdom of Saudi Arabia*

## Abstract

Cerebral palsy (CP) is a common chronic motor disorder with associated cognitive, communicative, and seizure disorders. Children with CP have a higher risk of dental problems creating significant morbidity that can further affect their wellbeing and negatively impact their quality of life. Screening for dental disease should be part of the initial assessment of any child with CP. The objective of this article is to present an updated overview of dental health issues in children with CP and outline important preventative and practical strategies to the management of this common comorbidity. Providing adequate oral care requires adaptation of special dental skills to help families manage the ongoing health issues that may arise. As oral health is increasingly recognized as a foundation for general wellbeing, caregivers for CP patients should be considered an important component of the oral health team and must become knowledgeable and competent in home oral health practices.

Cerebral palsy (CP) is a common pediatric disorder occurring in approximately 2-2.5 per 1000 live births.^1^ It is a chronic motor disorder resulting from a non-progressive (static) insult to the developing brain.^2^ The motor disorders associated with CP are often accompanied by disturbances in coordination, cognition, communication, and seizure disorders.^3^,^4^ Children with CP are at increased risk of developing dental problems as compared with healthy controls.^5^ This can create significant morbidity that can further affect the wellbeing of these compromised children and negatively impact their quality of life.^6^ Screening for these conditions should be part of the initial assessment. The objectives of this article are to present an updated overview of dental health issues in children with CP and outline important preventative and practical strategies to the management of this common comorbidity.

## Predisposition to dental disease in CP

Studies have shown that the more severe the neurological insult in children with CP, the higher is the risk of dental disease.^7^,^8^ This results from multiple factors including motor and coordination difficulties, as well as limited oral care and hygiene. Various possible predisposing factors are summarized in **Table 1**. These include mental retardation, which is more common in children with severe CP particularly in those with epilepsy or cortical abnormalities on neuroimaging.^9^ Children with mental retardation are dependent on their caregiver for maintaining oral and dental hygiene making them at higher risk for dental disease. In addition, approximately 30% of CP patients are undernourished, affecting their dental health.^10^ The leading cause of poor nutrition appears to be pseudo-bulbar palsy, affecting the coordination of sucking, chewing, and swallowing. Excessive drooling (sialorrhea) also results from pseudo-bulbar palsy, however, it may also be related to increased production of saliva secondary to an irritating oral lesion, such as dental caries or infection.^11^ In addition, gastroesophageal reflux disease (GERD) is another common problem in children with CP causing regurgitation, vomiting, and possible aspiration.^12^ The GERD affects the dental health and results in dental erosions.^13^

**Table 1:** Factors possibly predisposing to dental disease in children with cerebral palsy.

Predisposing factors	Mechanism
Motor weakness or incoordination	Inability to maintain oral hygiene Depending on a caregiver for self care risk of dental trauma
Mental retardation	Inability to maintain oral hygiene Depending on a caregiver for self care
Pseudo-bulbar palsy	Chewing and swallowing difficulties Risk of dental caries and erosions Excessive drooling (sialorrhea)
Gastroesophageal reflux disease	Recurrent regurgitation and vomiting causing dental erosions
Malnutrition	Poor calcium intake Vitamin D deficiency

## Specific dental manifestations: Dental Caries

In general, many factors contribute to the development of dental caries including biological, economic, cultural, environmental and social factors.^14^ Patients with CP are at increased risk of developing dental caries affecting negatively their quality of life.^15^ Children with more severe neurological insult are at a greater the risk.^16^ The degree of cognitive and motor deficits is directly proportional to the likelihood of developing dental caries.^17^ Severe motor incoordination affects the ability to perform adequate oral hygiene and cognitive deficits makes cooperation for effective oral care more difficult.^18^

## Periodontal disease

Several studies have shown that gingival hyperplasia and associated bleeding occurs with higher frequency in children with CP.^19^,^20^ This high frequency may be due to the same factors predisposing to dental caries and leading to biofilm buildup.^21^ Difficulties in conducting daily oral hygiene, intraoral sensitivity, and oro-facial motor dysfunction are the main contributing factors.^22^ Another important factor is the use of antiepileptic drugs, particularly phenytoin.^23^ Gingival hyperplasia is predictive for periodontal diseases. It tends to occur in children with spastic quadriplegic CP, particularly with advancing age. Choreothetoid CP may also be associated with periodontal disease as a result of the continuous uncontrolled movements of the head making oral hygiene more difficult.^24^

## Dental erosion

Dental erosion is a progressive loss of hard dental tissue resulting from a chemical (non-bacterial) process.^25^ Gastroesophageal reflux disease is the single most important cause of dental erosions noted in up to 55% of patients.^26^ In one study, 75% of children with reflux on a 24-hour esophageal pH monitoring had moderate to severe erosion.^27^ Dental erosion is common in patients with CP who are predisposed to GERD. Another study found 73% of CP patients with dental erosions had history of GERD.^28^ Swallowing difficulties and recurrent chest infections were associated with the development of dental erosion in another study.^29^ Enamel erosion that affects the posterior dentition may be the first indication of GERD. However, both primary and permanent teeth can be affected, most commonly the upper molars, lower molars and upper incisors. Continuous chemical exposure may gradually result in the extension of the dental erosions. Early effective treatment of GERD is critical to avoid irreversible dental damage.^30^ Prevention, early identification, and intervention are needed to prevent permanent damage.

## Sialorrhea

Drooling of saliva (sialorrhea) appears to be the consequence of a dysfunction in the coordination of swallowing mechanisms (pseudo-bulbar palsy) and mouth opening. Drooling is not socially accepted and can produce significant negative effects on the psychosocial health and quality of life.^31^ It occurs in up to 30% of children with CP.^32^ Sometimes drooling is related to an irritating lesion, such as dental caries or throat infection, resulting in increased production of saliva. Severe drooling may get worse with some antiepileptic drugs, such as clonazepam, leading to aspiration syndrome, skin irritation, and articulation difficulties.^32^ Management of this difficult problem is not very effective and includes a trial of an anticholinergic medication, such as glycopyrrolate and scopolamine. Side effects include irritability, sedation, blurred vision, and constipation.^33^ Scopolamine is also available as a skin patch. Surgical re-routing of salivary ducts is an option, however, it may lead to increased aspiration.^33^ Botulinum toxin injection into the parotid and submandibular glands may be effective in reducing excessive drooling.^34^

## Bruxism

Bruxism, the habitual grinding of teeth, is a common problem in children with CP, particularly those with severe motor and cognitive deficits.^35^ Bruxism may lead to teeth abrasion and flattening of biting surfaces. The exact mechanisms causing the development of this habit is not fully known, however, it is likely a self-stimulatory behavior and could also be related to abnormal proprioception in the periodontium.^36^ It is known that children with CP are predisposed to such abnormal behaviors including finger sucking and other mouthing habits. Local dental factors, such as malocclusion, should be excluded. As well, sleep disorders may predispose to the development of nocturnal bruxism, particularly in those with severe visual impairment.^37^ Disturbed and fragmented sleep is very disruptive to the parents as a result of frequent nocturnal awakenings. Medications that improve the sleep-wake cycle, such as melatonin, should be used and may also result in improved daytime behavior.^37^

## Traumatic dental injuries

Motor deficits and epilepsy increase the risk of physical injuries in children with CP. Malocclusion with prominent maxillary incisors and incompetent lips represent local risks that further predisposing to dental trauma.^38^ The risk varies between 10-20% and can reach 60% in patients with drop attacks.^39^ In addition to facial injury, these children are predisposed to fracture of enamel and dentine.^40^

## Malocclusion

Malocclusion has been reported with increasing frequency in children with CP, most commonly over-bite and anterior open-bite.^41^ These abnormalities have been reported to get worse with age.^42^ Mouth breathing, lip incompetence and long face are contributing factors.^43^ Pseudo-bulbar palsy, oro-facial incoordination and hypotonia could further add to the risk of developing malocclusion.

## Enamel defects

Children with CP are at an increased risk for having developmental enamel defects.^44^ Around 40% of affected children were born prematurely (<37 weeks). These enamel defects are located in a symmetrical manner in both primary incisors and first molars.

## Temporomandibular joint (TMJ) disorders

Children with CP are at a significantly higher risk for developing signs and symptoms of TMJ disorders.^45^ Male gender, the presence and severity of any malocclusion, mouth breathing, and mixed dentition were all identified as risk factors for developing signs and symptoms of TMJ disorders in CP patients.

## Dental management

Some practical challenges are commonly encountered when handling children with CP. These include apprehension, fear from strangers, and communication difficulties.^46^ Effective communication with such children during dental assessment should take in consideration their developmental age and any associated auditory, visual or speech disorders. Cognitive and attention deficits can also contribute to cooperation difficulties. Special seating and positioning adjustments are needed for children with abnormal posture. The dental chair should allow careful adjustment to provide the needed stability and support. Tipping the chair well back is often needed in spastic and athetoid CP patients with more manual control. Supportive and relaxed approach can help in improving the child’s cooperation.^46^ A useful tip is to schedule the visit early in the day and allow sufficient time to establish appropriate interaction during such encounters.^47^ The dentist may not establish much during the first visit that may be used mainly to establish mutual confidence and have a preliminary assessment. Assistance from the parents and dental assistant is often needed particularly for immobilization and during X-ray procedures. Patients with more severe spasticity involving the head and neck may be best evaluated on the parent’s lap.^48^ Head position can be also maintained in the midline by the help of Velcro straps. Open mouth can be maintained with the use of mouth props and the dentist should try their best to be gentle, caring, and avoid sudden movements that may trigger muscle spasm or stiffening. A finger guard and a steel mirror are preferred to avoid injury or shattering. Sharp instruments should be used with extreme caution to prevent injury. There are no reservations on using local anesthesia. CP patients often have difficulty rinsing appropriately necessitating the provision of water spray and suction device. Orthodontic or prosthetic parts are advisable only if the disability is mild to minimize the risk of breakage and aspiration.

## Sedation & anesthesia

Children with CP may be difficult to handle and uncooperative during dental assessment and management. Sedation and anesthesia is frequently needed in such situations, particularly if invasive procedures are needed.^49^ History of respiratory difficulties and seizures represent a particular challenge. Assessment by the concerned specialty (pediatrics, anesthesia, and/or neurology) is often needed prior to the required procedure. If the procedure is associated with prolonged period of decreased oral intake, intravenous antiepileptic drugs can replace the oral medications. Drugs like phenobarbitone or phenytoin can be used, however, a loading dose should be initiated before the procedure for optimal effects.^50^ Once the patient is able to take the oral drugs, IV drugs can be weaned quickly.

Many drugs can be used to induce sedation and anesthesia including benzodiazepines, nitrous oxide, narcotics, and propofol.^51^ Most children with CP and severe mental disability do not tolerate initial facemask prior to IV sedation. However, nasal or facemask can be utilized in milder cases to avoid the fear and anxiety associated with IV insertion. Oxygen saturation should be monitored by pulse oximetry and the airway should be protected throughout the procedure. Children with CP are at an increased risk of aspirating dental filling materials, debris from preparation of the tooth, or even an extracted tooth. This is in addition to excessive salivation and water spray used for cooling instruments.^51^ A throat shield should always be used to further protect the airway in these cases. Postoperative care include keeping the child with CP restrained until he or she is able to respond to verbal commands or become fully consciousness. IV cannulas and monitor should be removed as soon as possible as they add to the child’s fear and anxiety. Most patients with CP tolerate such procedures and sedation well with minimal postoperative complications.^52^

## Prevention

Home dental care and hygiene should be promoted from early on. Parents should learn to start gently daily cleansing of the incisors with a soft cloth or an infant soft toothbrush. For older children who are unwilling or physically unable to cooperate, the dentist should teach the parent proper brushing techniques and ways to safely restrain the child when necessary. The child is placed in the parent’s lap to stabilized the head with one hand while using the other hand to brush the teeth. An older child may recline on a chair or bed and the parent angles the head backward with one hand while the teeth are brushed with the other hand. More extreme restraining by both parents is needed for the more difficult child.^53^ The patient’s hands may have to be restrained by a second or third person for effective oral cleansing.^53^ To encourage independence of children with milder motor disabilities, an electric toothbrush may be utilized effectively.

In conclusions, as oral health is increasingly recognized as a foundation for general wellbeing, caregivers for CP patients should be considered an important component of the oral health team and must become knowledgeable and competent in home oral health practices. Such practices can significantly affect the child’s quality of life and control dental costs.
